# Development and external validation of the DOAT and DOATS scores: simple decision support tools to identify disease progression among nonelderly patients with mild/moderate COVID-19

**DOI:** 10.1186/s12890-023-02604-3

**Published:** 2023-08-28

**Authors:** Yoko Shibata, Kenji Omae, Hiroyuki Minemura, Yasuhito Suzuki, Takefumi Nikaido, Yoshinori Tanino, Atsuro Fukuhara, Ryuzo Kanno, Hiroyuki Saito, Shuzo Suzuki, Taeko Ishii, Yayoi Inokoshi, Eiichiro Sando, Hirofumi Sakuma, Tatsuho Kobayashi, Hiroaki Kume, Masahiro Kamimoto, Hideko Aoki, Akira Takama, Takamichi Kamiyama, Masaru Nakayama, Kiyoshi Saito, Koichi Tanigawa, Masahiko Sato, Toshiyuki Kambe, Norio Kanzaki, Teruhisa Azuma, Keiji Sakamoto, Yuichi Nakamura, Hiroshi Ohtani, Mitsuru Waragai, Shinsaku Maeda, Tokiya Ishida, Keishi Sugino, Minoru Inage, Noriyuki Hirama, Kodai Furuyama, Shigeyuki Fukushima, Hiroshi Saito, Jun-ichi Machiya, Hiroyoshi Machida, Koya Abe, Katsuyoshi Iwabuchi, Yuji Katagiri, Yasuko Aida, Yuki Abe, Takahito Ota, Yuki Ishizawa, Yasuhiko Tsukada, Ryuki Yamada, Riko Sato, Takumi Onuma, Hikaru Tomita, Mikako Saito, Natsumi Watanabe, Mami Rikimaru, Takaya Kawamata, Takashi Umeda, Julia Morimoto, Ryuichi Togawa, Yuki Sato, Junpei Saito, Kenya Kanazawa, Noriaki Kurita, Ken Iseki

**Affiliations:** 1 Department of Pulmonary Medicine, Fukushima Medical University, Fukushima, Japan; 2 Department of Innovative Research and Education for Clinicians and Trainees, Fukushima Medical University Hospital, Fukushima, Japan; 3 Department of Pulmonary Medicine, Ohara General Hospital, Fukushima, Japan; 4 Department of Thoracic Surgery, Fukushima Red Cross Hospital, Fukushima, Japan; 5 Department of Internal Medicine, Fujita General Hospital, Date-gun, Japan; 6 Department of Pulmonary Medicine, Saiseikai Fukushima General Hospital, Fukushima, Japan; 7 Department of General Internal Medicine and Clinical Infectious Diseases, Fukushima Medical University, Fukushima, Japan; 8 Department of General Internal Medicine and Infectious Diseases, Kita-Fukushima Medical Center, Date-shi, Japan; 9 Department of Internal Medicine, Saiseikai Kawamata Hospital, Kawamata, Japan; 10 Department of Emergency and Critical Care Medicine, Aizu Chuo Hospital, Aizuwakamatsu, Japan; 11 Department of Infectious Disease and Pulmonary Medicine, Aizu Medical Center, Fukushima Medical University, Aizuwakamatsu, Japan; 12 Department of Internal Medicine, Takeda General Hospital, Aizuwakamatsu, Japan; 13 Department of Pediatric Medicine, Bange Kousei General Hospital, Kawanuma, Japan; 14 Department of Surgery, Yurin Hospital, Kitakata, Japan; 15 Department of Pediatric Surgery, Iwaki City Medical Center, Iwaki, Japan; 16 Department of Internal Medicine, Kashima Hospital, Iwaki, Japan; 17 Department of Neurosurgery, Fukushima Rosai Hospital, Iwaki, Japan; 18 Department of Emergency and Critical Care Medicine, Futaba Medical Center, Futaba, Japan; 19 Department of Internal Medicine, Soma General Hospital, Soma, Japan; 20 Department of Pulmonary Medicine, Minami-Soma Municipal General Hospital, Minami-Soma, Japan; 21 Department of Surgery, Onahama Chuo Clinic, Iwaki, Japan; 22 Department of General Medicine, Shirakawa Satellite for Teaching and Research, Fukushima Medical University, Shirakawa, Japan; 23 Department of Cardiology and Vascular Medicine, Hoshi General Hospital, Koriyama, Japan; 24 Department of Internal Medicine, Iwase General Hospital, Sukagawa, Japan; 25 Department of Surgery, Southern TOHOKU General Hospital, Koriyama, Japan; 26 Department of Pulmonary Medicine, Jusendo General Hospital, Koriyama, Japan; 27 Department of Emergency and Critical Care Medicine, Ohta Nishinouchi Hospital, Koriyama, Japan; 28 Department of Respiratory Medicine, Tsuboi Hospital, Koriyama, Japan; 29 Department of Pulmonary Medicine, Okitama General Hospital, Higashi-Okitama, Japan; 30 Department of Pulmonary Medicine, Nihonkai General Hospital, Sakata, Japan; 31 Department of Pulmonary Medicine, Yamagata City Hospital Saiseikan, Yamagata, Japan; 32 Department of Emergency and Critical Care Medicine, Fukushima Medical University, Fukushima, Japan

**Keywords:** COVID-19, Disease deterioration, Nonelderly, Risk factor

## Abstract

**Background:**

During the fifth wave of the coronavirus disease 2019 (COVID-19) pandemic in Japan, which took place between June and September 2021, a significant number of COVID-19 cases with deterioration occurred in unvaccinated individuals < 65 years old. However, the risk factors for COVID-19 deterioration in this specific population have not yet been determined. This study developed a prediction method to identify COVID-19 patients < 65 years old who are at a high risk of deterioration.

**Methods:**

This retrospective study analyzed data from 1,675 patients < 65 years old who were admitted to acute care institutions in Fukushima with mild-to-moderate-1 COVID-19 based on the Japanese disease severity criteria prior to the fifth wave. For validation, 324 similar patients were enrolled from 3 hospitals in Yamagata. Logistic regression analyses using cluster-robust variance estimation were used to determine predictors of disease deterioration, followed by creation of risk prediction scores. Disease deterioration was defined as the initiation of medication for COVID-19, oxygen inhalation, or mechanical ventilation starting one day or later after admission.

**Results:**

The patients whose condition deteriorated (8.6%) tended to be older, male, have histories of smoking, and have high body temperatures, low oxygen saturation values, and comorbidities, such as diabetes/obesity and hypertension. Stepwise variable selection using logistic regression to predict COVID-19 deterioration retained comorbidities of diabetes/obesity (DO), age (A), body temperature (T), and oxygen saturation (S). Two predictive scores were created based on the optimism-corrected regression coefficients: the DOATS score, including all of the above risk factors, and the DOAT score, which was the DOATS score without oxygen saturation. In the original cohort, the areas under the receiver operating characteristic curve (AUROCs) of the DOATS and DOAT scores were 0.81 (95% confidence interval [CI] 0.77–0.85) and 0.80 (95% CI 0.76–0.84), respectively. In the validation cohort, the AUROCs for each score were both 0.76 (95% CI 0.69–0.83), and the calibration slopes were both 0.80. A decision curve analysis confirmed the clinical practicability of both scores in the validation cohort.

**Conclusions:**

We established two prediction scores that can quickly evaluate the risk of COVID-19 deterioration in mild/moderate patients < 65 years old.

**Supplementary Information:**

The online version contains supplementary material available at 10.1186/s12890-023-02604-3. The calculator of the DOATS and DOAT scores are available at https://shibatay8.wixsite.com/doats-score--a-simpl/post/doats-score.

## Introduction

Due to the global spread of severe acute respiratory syndrome coronavirus 2 (SARS-CoV-2), the coronavirus disease 2019 (COVID-19) pandemic remains a serious problem worldwide. Approximately 5% of COVID-19 patients develop respiratory failure, and 2% die despite undergoing intensive treatment [1]. During the height of the pandemic, many patients in Japan could not be admitted to hospitals due to the lack of available beds in urban areas such as Tokyo, Kobe, and Osaka. Therefore, it is important to predict which patients will become severely ill as early as possible after the onset, even in those without respiratory failure. In particular, the administration of antiviral drugs and neutralizing antibodies is considered most effective when performed within five to seven days after the onset [2].

Some observational studies have revealed the risk factors for severe COVID-19. Old age, male sex, a smoking habit, and comorbidities such as diabetes, chronic renal disease, malignancies, and chronic respiratory diseases, such as chronic pulmonary disease (COPD) or interstitial lung diseases, have been listed as risk factors for severe COVID-19 [3–8]. In addition, some biomarkers have been reported to be associated with disease severity [4, 8–12]. Using these risk factors, several investigators have reported risk stratification methods [9–11, 13–16].

Beginning in February 2021, vaccines for SARS-CoV-2 began to be disseminated in Japan. By the end of July 2021, 30% of residents in Japan had been vaccinated twice. A majority of people who had been vaccinated twice were elderly individuals (≥ 65 years old) or health-care workers. The widespread dissemination of highly effective vaccines has resulted in a reduction in COVID-19 incidence and a decrease in the severity of cases among vaccinated individuals ≥ 65 years old in Japan [17]. Accordingly, the next target population for health care policy has shifted to individuals < 65 years old who have not yet received the vaccine [17]. It is difficult to accurately forecast the risk for disease deterioration by applying existing prediction rules based on risk factors identified before the dissemination of vaccines to these younger target populations under the current conditions. However, few investigations have reported on the risk factors among the general population < 65 years old who have not been vaccinated for SARS-CoV-2 [18].

The present study therefore investigated the risk factors for COVID-19 deterioration among patients < 65 years old who had not received the vaccine and did not require oxygen supplementation, i.e. those with mild-to-moderate-1 stage of COVID-19 according to the Japanese disease severity criteria [19], and developed and externally validated clinical prediction rules to identify cases at risk of deterioration. In particular, we analyzed the factors associated with disease deterioration, including not only death and the initiation of mechanical ventilation but also the start of medications such as remdesivir or dexamethasone and the start of oxygen inhalation during hospitalization.

## Methods

### Setting and study population

This multicenter retrospective cohort study used the data of all consecutive patients with COVID-19 admitted to the hospitals that participated in a web conference against COVID-19 held weekly by the Department of Pulmonary Medicine, Fukushima Medical University, between March 31, 2020, and May 20, 2021. Of the 43 COVID-19 hospitals in Fukushima, 26 facilities that engaged in medical care during the acute phase of the disease participated in this conference. Of approximately 4,500 patients with COVID-19 in Fukushima, 3,008 (67%) were enrolled in this study. For the external validation study, 3 hospitals in Yamagata treating COVID-19 patients between March 31, 2020, and May 20, 2021, provided data on 324 mild and moderate-1 patients < 65 years old. A diagnosis of COVID-19 was made when the results of a polymerase chain reaction (PCR) test using a nasopharyngeal swab or saliva were positive.

The subjects’ data, such as clinical characteristics including comorbidities, results of examinations, medical course, medications, and outcomes, were obtained from the medical records of each hospital. COVID-19 disease severity was classified according to the definition of the Japanese Ministry of Health, Labor and Welfare as follows: mild, subjects without pneumonia and respiratory failure; moderate-1, subjects with pneumonia but without respiratory failure; moderate-2, subjects with pneumonia and respiratory failure (oxygen saturation < 94% on room air) that does not require mechanical ventilation or extracorporeal membrane oxygenation (ECMO); severe, subjects with pneumonia and respiratory failure that required mechanical ventilation and ECMO [19, 20]. In this article, the term “moderate” refers to moderate-1 of the Japanese COVID-19 severity classification, unless otherwise specified.

**Fig. 1:**
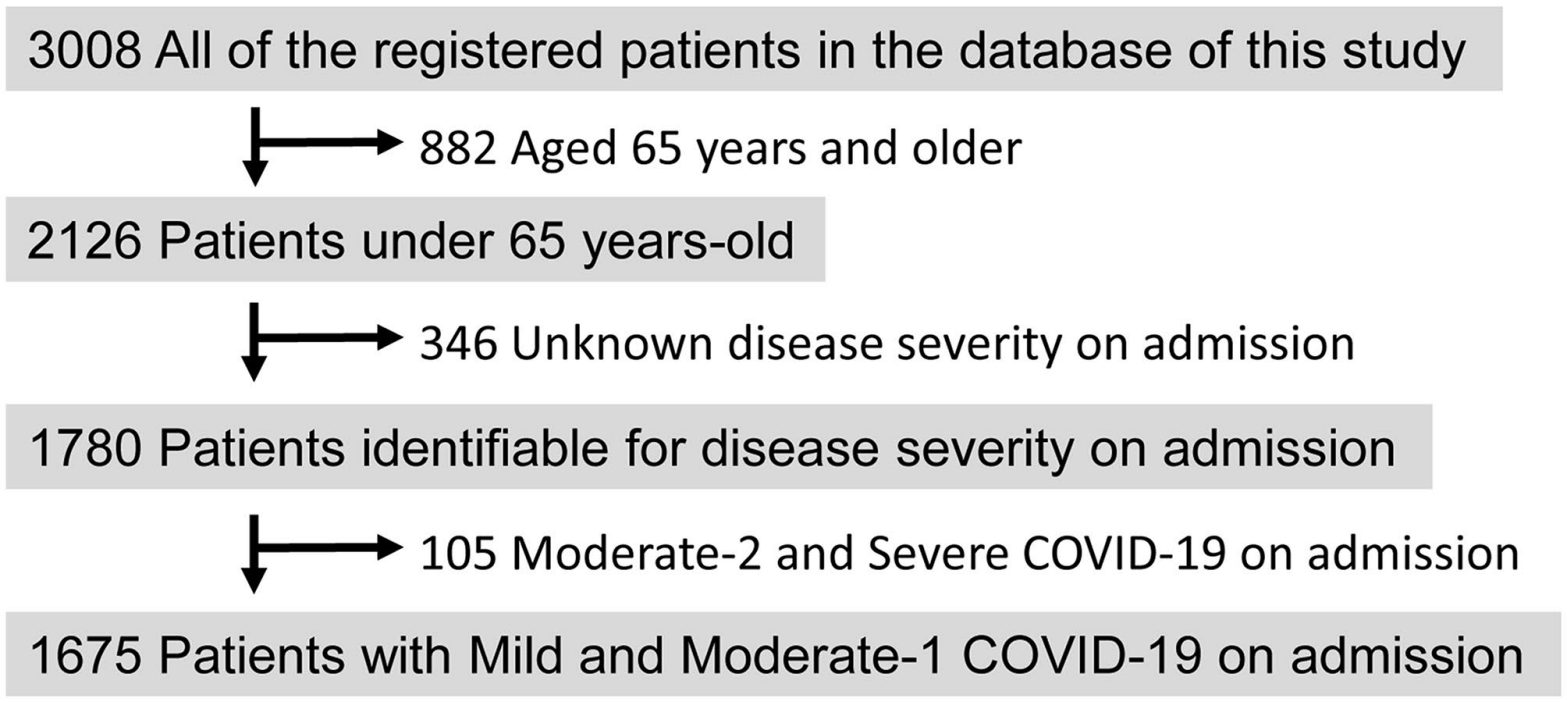
Flowchart of patient recruitment in this study. Among all registered inpatients with COVID-19 in Fukushima, 1,675 were selected for the present study. Disease severity was classified into mild, moderate-1, moderate-2, and severe in accordance with the definition of the Japanese Ministry of Health, Labor and Welfare, as described in the *Methods* section of this manuscript

Among the 3,008 enrolled patients, 1,675 from 21 facilities had mild and moderate-1 disease and were < 65 years old. These were the eligible subjects in this study (Fig. 1, Supplementary Table 1).

### Outcomes

The outcomes were (1) any disease deterioration, defined as the initiation of medication for COVID-19 (dexamethasone, methylprednisolone, tocilizumab, baricitinib, or remdesivir) or respiratory therapy (use of inhalation oxygen); or (2) use of a ventilator or the need for ECMO after the first day of hospitalization.

### Candidate predictors

The age, sex, body temperature on admission (< 37.0, 37.0-37.9, ≥ 38.0 °C), history of smoking, comorbidities (prevalence ≥ 2%), pregnancy, and blood oxygen saturation (%) measured by a pulse oximeter on admission were evaluated as candidate risk factors for deterioration of the disease [21–23]. The collection of information was left to the doctors at each participating institution.

### Statistical analyses

Continuous variables are presented as medians with interquartile ranges, and categorical variables are presented as the number of patients with percentages. Patients whose conditions were exacerbated a day or later after admission were assigned to the Deteriorated group, while all others were assigned to the Stable group. Between-group comparisons were performed using the Mann‒Whitney U test for continuous variables and the chi-square test for categorical variables.

The variables with statistically significant differences between the two groups were used to identify possible risk factors for predicting the deterioration of COVID-19 a day or later after admission to the hospital by forward stepwise multivariate logistic regression analyses. In these analyses, cluster-robust variance estimation was used to consider within-hospital correlation [24]. Because information on oxygen saturation may not be available in some clinical sites, such as during patients’ temporary accommodations or at home, we analyzed two models: one including and one excluding oxygen saturation values. Among the comorbidities listed in the database from the participating hospitals in Fukushima, those with a prevalence of ≥ 2% were applied to the analyses. Diabetes and obesity were combined in the same category for the following reasons: first, obesity is an important risk factor for the onset and progression of diabetes, and second, because they both have common clinical features and immune responses to SARS-CoV-2 based on a background of chronic systemic low-grade inflammation, abnormal production of proinflammatory cytokines, and an impaired immune response and host defense [25], both are reported as high-risk factors for COVID-19 mortality [26]. For continuous variables, namely age and oxygen saturation, a restricted cubic spline curve with the logistic regression analysis confirmed the linear relationship with the risk of COVID-19 deterioration (Supplementary Fig. 1a and 1b).

We created the score-based prediction model with an emphasis on simplicity, prioritizing clinical usability with minimal burden on physicians [27]. First, age and oxygen saturation as continuous variables were incorporated into the model for the original cohort using logistic regression with clustering per hospital (original model). Next, a 200-cycle bootstrap process was performed to assess the optimism of the original model, and optimism-corrected regression coefficients for each variable were calculated (bootstrap shrinkage) [28]. The continuous variables were then categorized into 4 or 5 categories (i.e. 0–17, 18–30, 31–40, 41–50, and 51–64 for age [years old]; and 94%, 95%, 96-97%, and 98-100% for oxygen saturation), the midpoints for each category were determined, and “regression units” were calculated using the optimism-corrected regression coefficients, according to a previous study [27, 29]. Finally, points were assigned to each variable category, calculated by dividing the “regression units” by the lowest coefficient and rounding to the nearest integer.

We investigated the predictive accuracy of the models by testing discrimination and calibration in both the original and validation cohorts. Discrimination was assessed by calculating the area under the receiver operating characteristic curve (AUROC). We also presented predictive performance (sensitivity, specificity, and positive and negative likelihood ratios) stratified for each threshold of the scores. A calibration curve was generated to evaluate the agreement between the observed and predicted outcomes with respect to calibration ability. Furthermore, we evaluated the calibration slope and intercept (calibration-in-the-large) in the validation cohort. Finally, we used a decision curve analysis (DCA) to assess the clinical utility of the models [39].

A two-tailed p value of < 0.05 was considered statistically significant. Sample size calculations were not performed because we used all available data in the registry to maximize the power of the results. Cases with missing data were excluded from the analyses, and the details of the missing data are described in the note of Table 1. All statistical analyses were performed using the software programs JMP 13 (SAS Institute Inc, Cary, NC, USA), STATA 16 (Stata Corp LP, College Station, TX, USA), and SPSS27 (IBM Corp., Armonk, NY, USA).

**Table 1:** Differences in the characteristics between the Stable and Deteriorated groups in the original cohort of the study and external validation cohort

	Original cohort			External validation cohort		
	All	Stable group	Deteriorated group	All	Stable group	Deteriorated group
	n = 1675	n = 1531	n = 144	n = 324	n = 283	n = 41
Age, year	41 (25–53)	39 (24–52)	51 (41.25–59.75)***	42 (29.3–52)	41 (27–51)	51 (39.5–60.5)^###^
Male	919 (54.8%)	825 (53.9%)	94 (65.3%)***	181 (55.9%)	158 (55.8%)	23 (56.1%)^a^
Mild / Moderate-1	947/728	900/631	47/97 ***	260/64	240/43	20/21 ^###^
History of cigarette smoking	550 (38.0%)	479 (36.56%)	71 (52.59%)***	—	—	—
Temperature ≥ 37 °C	647 (40.4%)	549 (37.6%)	98 (68.5%)***	230 (71.0%)	195 (68.9%)	35 (85.4%)^#^
Temperature ≥ 38 °C	291 (17.8%)	219 (14.7%)	72 (50.7%)***	96 (29.6%)	73 (25.8%)	23 (56.1%)^###^
Diabetes or Obesity	175 (11.2%)	132 (9.26%)	43 (30.5%)***	53 (16.6%)	41 (14.7%)	12 (29.3%)^#^
Hypertension	197 (12.6%)	155 (10.87%)	42 (29.8%)***	—	—	—
Chronic respiratory diseases	75 (4.8%)	67 (4.7%)	8 (5.7%)	—	—	—
Malignancies	37 (2.4%)	31 (2.2%)	6 (4.3%)	—	—	—
Dyslipidemia	80 (5.1%)	68 (4.74%)	12 (8.51%)	—	—	—
Cardiac diseases	40 (2.5%)	33 (2.30%)	7 (4.96%)	—	—	—
Pregnancy	13 (0.8%)	13 (0.8%)	0 (0%)	—	—	—
MV and/or ECMO	7 (0.4%)	0 (0%)	7 (0.4%)	—	—	—
Deceased due to COVID-19	1 (0.06%)	0 (0%)	1 (0.06%)	—	—	—
SpO_2_, %	97 (97–98)	98 (97–98)	97 (96–97.75)***	97 (96–98)	97 (97–98)	97 (96–98)^b^

***: *P* < 0.0001 vs. Stable group (original cohort), ###: *P* < 0.0001 vs. Stable group (validation cohort), #: *P* < 0.05 vs. Stable group (validation cohort), a: *P* = 0.974 vs. Stable group (validation cohort), b: *P* = 0.055 vs. Stable group (validation cohort)

In the original cohort, the numbers of missing data regarding “a history of cigarette smoking”, “temperature ≥ 37°C”, “temperature ≥ 38°C”, “diabetes or obesity”, “hypertension”, “chronic respiratory disease”, “malignancies”, “dyslipidemia”, “cardiac diseases”, and “SpO_2_” were 230, 73, 38, 108, 108, 99, 99, 99, 99, and 6, respectively

In the external validation cohort, the numbers of missing data regarding “diabetes or obesity” was four

Coronavirus disease 2019, COVID-19; extracorporeal membrane oxygenation, ECMO; mechanical ventilation, MV

## Results

Table 1 shows the characteristics of the mild/moderate COVID-19 patients < 65 years old in the current study. One hundred and forty-four patients (8.6%) experienced deterioration of their disease 1 day after admission or later. These patients were older and more likely to be male with histories of cigarette smoking, a higher body temperature, lower oxygen saturation, and higher rates of comorbidities, such as diabetes/obesity and hypertension, than the Stable group. The rates of pregnancy, as well as comorbidities, such as chronic respiratory diseases, malignancies, dyslipidemia, and cardiac diseases, were not significantly different between the Deteriorated and Stable groups.

**Fig. 2:**
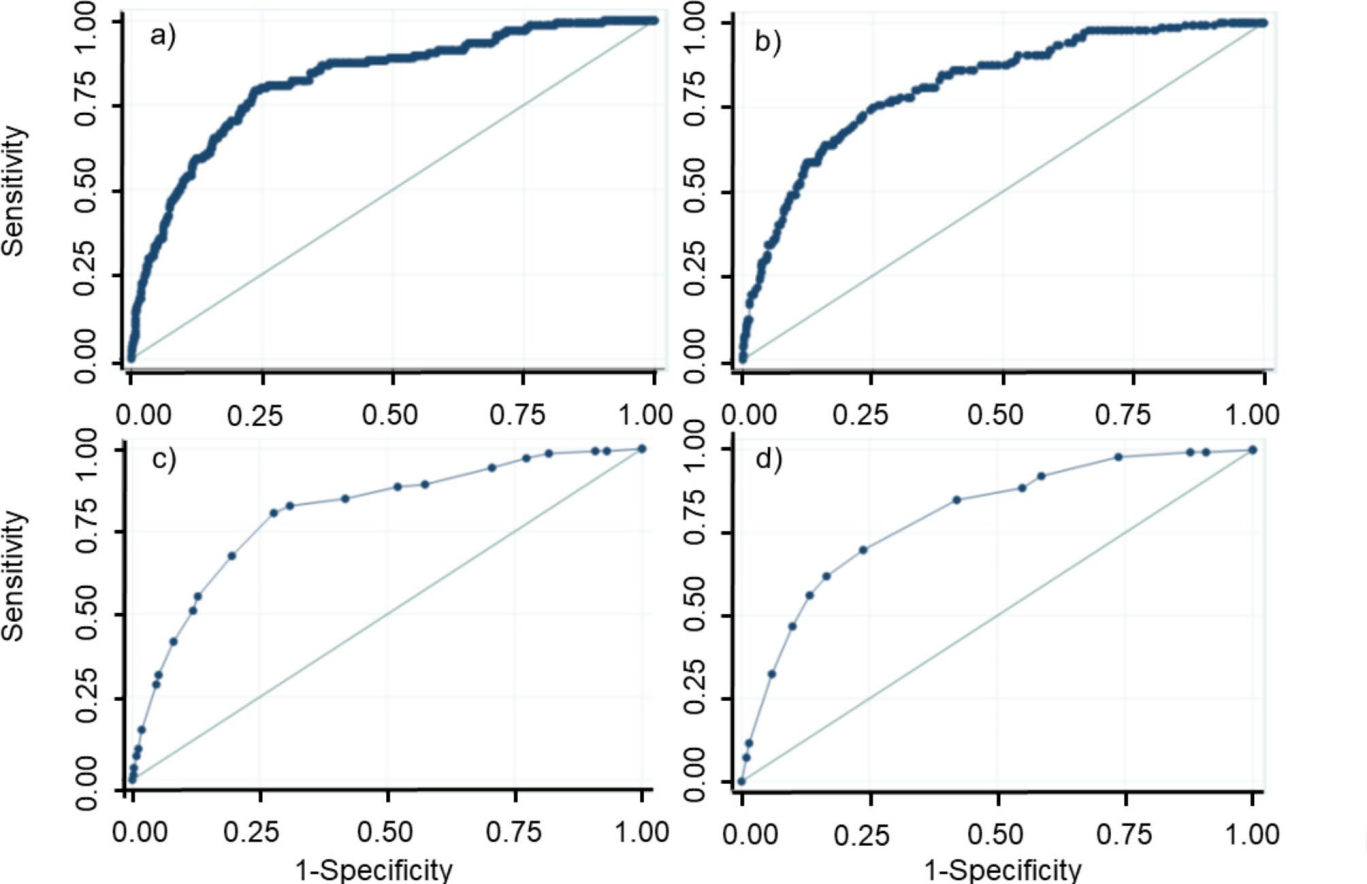
Receiver operating characteristic (ROC) curves analyzing the discrimination of (**a**) the original model with DOATS, (**b**) the original model with DOAT, (**c**) the DOATS score, and (**d**) the DOAT score for COVID-19 deterioration in the original cohort. Of the original models that included continuous variables as they were, the optimism-corrected area under the ROC curves (AUROCs) for (**a**) the DOATS-based and (**b**) DOAT-based models predicting the deterioration of COVID-19 patients during hospitalization were 0.82 (95% confidence interval [CI] 0.77–0.85) and 0.81 (95% CI 0.77–0.86), respectively. For (**c**) the DOATS and (**d**) DOAT scores, the AUROCs were 0.81 (95% CI 0.77–0.85) and 0.80 (95% CI 0.76–0.84), respectively

**Fig. 3:**
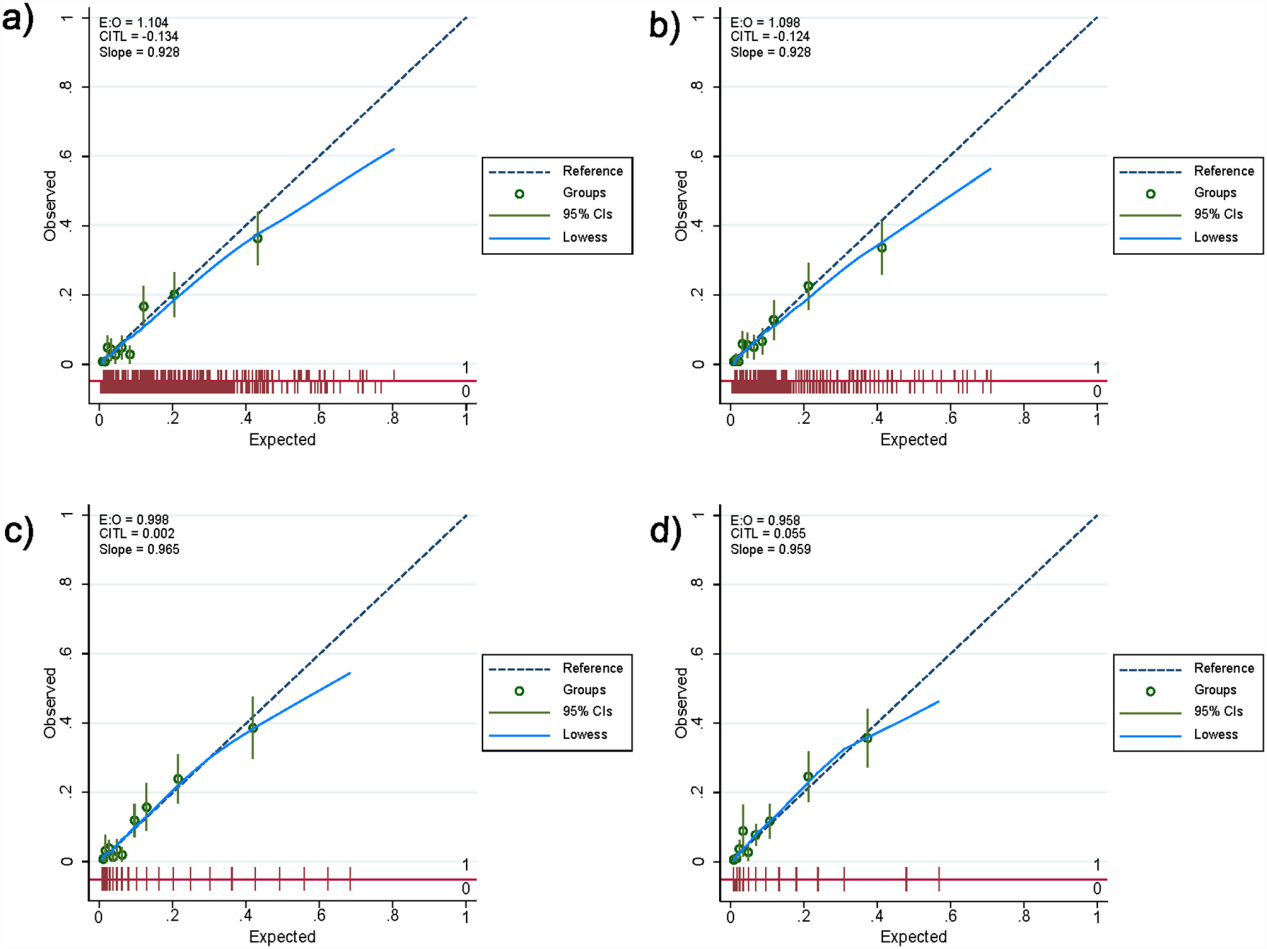
Calibration curves of (**a**) the original model with DOATS, (**b**) the original model with DOAT, (**c**) DOATS score and (**d**) DOAT score for COVID-19 deterioration in the original cohort. The calibration curve analysis showed that the calibration slope and calibration-in-the-large were 0.93 and 0.134 for the original DOATS model (**a**), 0.93 and 0.124 for the original DOAT model (**b**), 0.97 and 0.002 for the DOATS score (**c**), and 0.96 and 0.055 for the DOAT score (**d**), respectively. The calibration curves of the original models (**a**, **b**) and the scores (**c**, **d**) demonstrated good agreement between the predicted probability of COVID-19 deterioration and the observed COVID-19 deterioration

Stepwise variable selection using a logistic regression analysis with clustering per hospital to predict COVID-19 deterioration during hospitalization in the original cohort retained comorbidities of diabetes/obesity (DO), age (A), body temperature (T), and oxygen saturation (S). Table 2 shows the results of the multivariable logistic regression analysis including all of the above risk factors (DOATS) and all but oxygen saturation (DOAT) before and after optimism correction with 200 cycles of bootstrapping. Of the original models that included continuous variables as they were, the optimism-corrected AUROCs for the DOATS-based and DOAT-based models predicting deterioration in COVID-19 patients during hospitalization were 0.82 (95% confidence interval [CI] 0.77–0.85) and 0.81 (95% CI 0.77–0.86), respectively (Fig. 2a and b). The calibration curve showed good agreement between the predicted probability of COVID-19 deterioration by the original models and the observed COVID-19 deterioration (Fig. 3a and b).

**Table 2:** Results of a multivariable logistic regression analysis with all risk factors (DOATS) and with all but oxygen saturation (DOAT) before and after optimism correction with 200 cycles of bootstrapping in the original cohort

Variable	DOATS-based original model					DOAT-based original model			
	Before optimism correction		After optimism correction			Before optimism correction		After optimism correction	
	β coefficient	95% CI	β coefficient	95% CI		β coefficient	95% CI	β coefficient	95% CI
Intercept	23.407	9.458–37.356	21.921	21.731–22.111		-5.217	-5.882–-4.553	-5.136	-5.325–-4.947
Age, years	0.043	0.036–0.050	0.041	0.034–0.047		0.049	0.041–0.058	0.046	0.038–0.055
Temperature, °C									
37.0–37.9	0.283	-0.280–0.847	0.268	-0.266–0.801		0.381	-0.168–0.931	0.359	-0.159–0.877
≥ 38.0	1.671	1.298–2.043	1.580	1.228–1.933		1.825	0.000–1.452	1.719	0.000–1.367
Diabetes or obesity	0.987	0.298–1.675	0.933	0.282–1.585		1.134	0.458–1.810	1.068	0.432–1.705
SpO2, %	-0.291	-0.435–-0.147	-0.275	-0.411–-0.139		ー	ー	ー	ー

confidence interval, CI; comorbidities of diabetes/obesity, age, body temperature, and oxygen saturation, DOATS; comorbidities of diabetes/obesity, age, and body temperature, DOAT

The DOATS and DOAT scores constructed by categorizing age and oxygen saturation and assigning score points to each category of final variables (DOATS and DOAT) based on optimism-corrected regression coefficients of the original models are listed in Table 3. Both scores were calculated for each patient by adding the points for risk factors present: the DOATS score ranges from − 2 to 19 points, while the DOAT score ranges from − 2 to 12 points. Table 4 shows the predicted probabilities of COVID-19 deterioration according to the scores along with the actual percentages of patients who had deterioration or required mechanical ventilation and/or ECMO treatment during hospitalization. Overall, the higher the points, the more deteriorated and the greater the need for mechanical ventilation/ECMO.

**Table 3:** (**a**) DOATS and (**b**) DOAT scores for COVID-19 deterioration based on optimism-corrected regression coefficients of the original models

	(a)						(b)					
Variable	Category	Midpoint^1^	Difference^2^	Coefficient	Regression unit^3^	Score	Category	Midpoint^1^	Difference^2^	Coefficient	Regression unit^3^	Score
Age, years				0.041						0.046		
	0–17	8.5	-15.5		-0.636	-2	0–17	8.5	-15.5		-0.713	-2
	18–30	24 (ref)	0		0	0	18–30	24 (ref)	0		0	0
	31–40	35.5	11.5		0.472	2	31–40	35.5	11.5		0.529	1
	41–50	45.5	21.5		0.882	3	41–50	45.5	21.5		0.989	3
	51–64	57.5	33.5		1.374	5	51–64	57.5	33.5		1.541	4
Temperature, °C												
	≤ 36.9			ref	0	0	≤ 36.9			ref	0	0
	37.0–37.9			0.268	0.268	1	37.0–37.9			0.359	0.359	1
	≥ 38.0			1.58	1.58	6	≥ 38.0			1.719	1.719	5
Diabetes or obesity												
	No			ref	0	0	No			ref	0	0
	Yes			0.933	0.933	3	Yes			1.068	1.068	3
SpO2, %				-0.275								
	94	94	-5		1.375	5						
	95	95	-4		1.1	4						
	96–97	96.5	-2.5		0.688	3						
	98–100	99 (ref)	0		0	0						

^1^Midpoint for each category of continuous variables

^2^Difference in each midpoint from the reference

^3^For continuous variables, each difference is multiplied by the regression coefficient for that predictor. For binary and categorical variables, the "regression units" are simply the regression coefficients for that predictor.

Coronavirus disease 2019, COVID-19; comorbidities of diabetes/obesity, age, body temperature, and oxygen saturation, DOATS; comorbidities of diabetes/obesity, age, and body temperature, DOAT

**Table 4:** Predicted and actual risk of COVID-19 deterioration, as well as the actual risk of using an MV and ECMO in the original cohort according to each (**a**) DOATS score and (**b**) DOAT score

(a)					(b)				
DOATS score	No.	Predicted risk (%)	Actual risk (%)	Actual risk of MV/ECMO (%)	DOAT score	No.	Predicted risk (%)	Actual risk (%)	Actual risk of MV/ECMO (%)
-2	95	0.8	1	0	-2	126	0.9	0.8	0
-1	31	1	0	0	-1	42	1.2	0	0
0	125	1.3	0.8	0	0	194	1.7	1	0
1	62	1.7	3.2	0	1	214	2.5	3.7	0
2	96	2.2	4.2	0	2	56	3.5	8.9	0
3	186	2.9	3.8	0	3	180	5	2.8	0
4	74	3.7	1.4	0	4	271	6.9	7.8	0
5	145	4.8	3.5	0	5	109	9.6	10.1	0
6	151	6.2	2	0	6	53	13.3	15.1	0
7	46	8	6.5	2.2	7	59	18	22	3.4
8	130	10.2	13.9	0	8	75	23.9	26.7	0
9	108	12.9	15.7	0	9	90	31	32.2	0
10	19	16.2	31.6	0	11	13	47.9	46.2	15.4
11	65	20.2	20	0	12	23	56.9	43.5	13
12	54	24.9	25.9	1.9					
13	10	30.2	40	10					
14	58	36.2	32.8	0					
15	17	42.6	47.1	5.9					
16	8	49.2	37.5	0					
17	12	55.9	41.7	8.3					
18	4	62.3	75	25					
19	5	68.4	40	20					

Coronavirus disease 2019, COVID-19; comorbidities of diabetes/obesity, age, body temperature, and oxygen saturation, DOATS; comorbidities of diabetes/obesity, age, and body temperature, DOAT; extracorporeal membrane oxygenation, ECMO; mechanical ventilation, MV

**Fig. 4:**
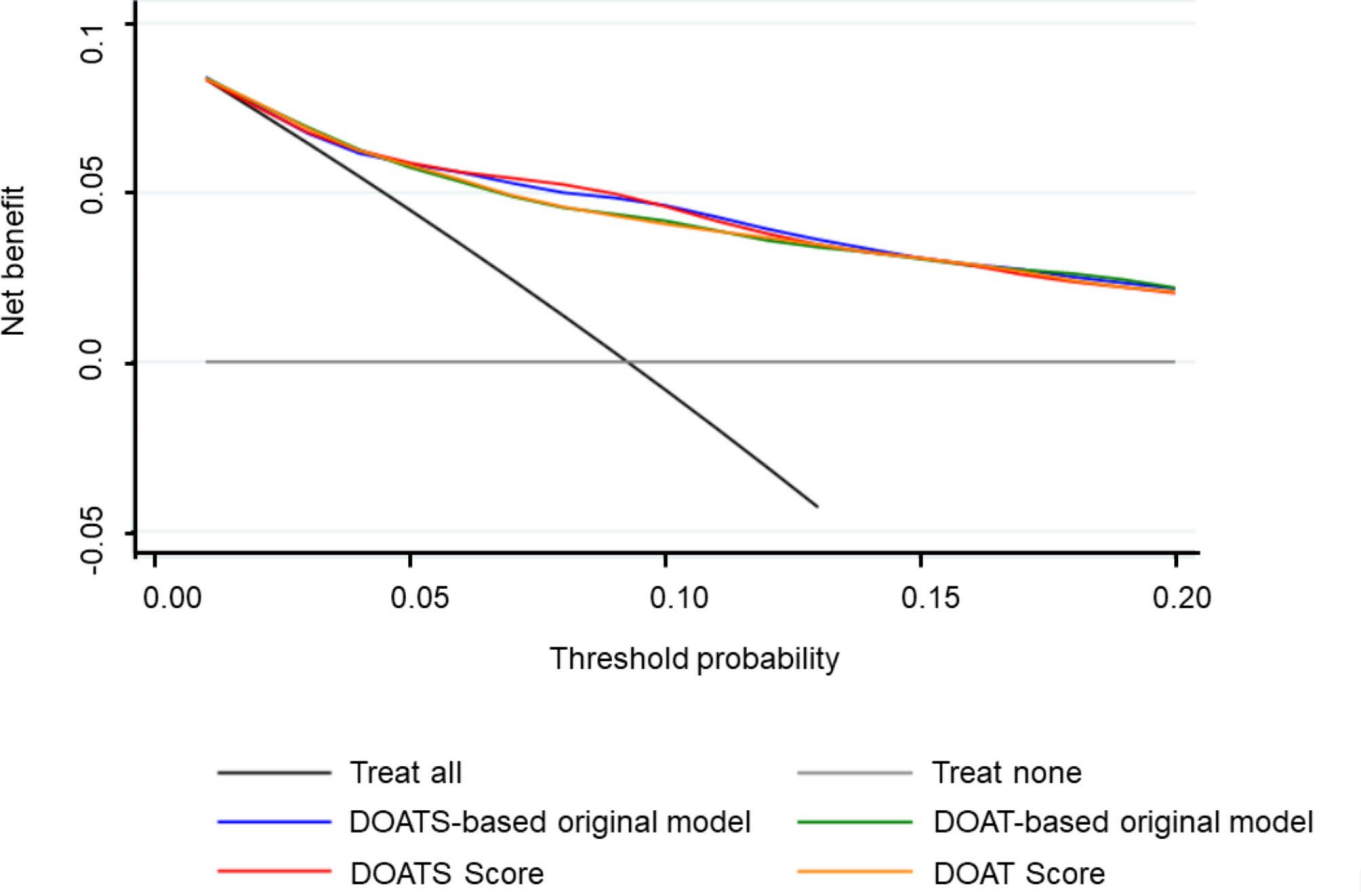
Decision curve analyses for the original model with DOATS, the original model with DOAT, and the DOATS and DOAT scores in the original cohort. The graph illustrates the net benefit relative to no treatment in any patient (‘Treat none’) using different treatment approaches. The gray line represents the scenario where no patients are treated, resulting in a net benefit of zero (no true-positive and no false-positive classifications). The black line represents the scenario where all patients are treated. The colored lines correspond to different treatment thresholds based on the predictions of the DOATS-based original model (blue line), DOAT-based original model (green line), DOATS score (red line), and DOAT score (orange line) for the risk of deterioration. This graph demonstrates the expected net benefit when treatment decisions are made based on these different approaches. The analysis confirms the clinical practicability and utility of both scores, as well as the original models

The prognostic performance of the DOATS and DOAT scores at each threshold is shown in Supplementary Table 2a and 2b. The AUROCs for the DOATS and DOAT scores predicting deterioration in COVID-19 patients during hospitalization were 0.81 (95% CI 0.77–0.85) and 0.80 (95% CI 0.76–0.84), respectively, resulting in only a small reduction from the AUROCs of the original models (Fig. 2c and d). Both of the predicted risks using these scores showed a generally good fit with the observed COVID-19 deterioration, as evidenced by the calibration curves (Fig. 3c and d). A DCA confirmed the clinical practicability of both scores as well as the original models (Fig. 4).

**Fig. 5:**
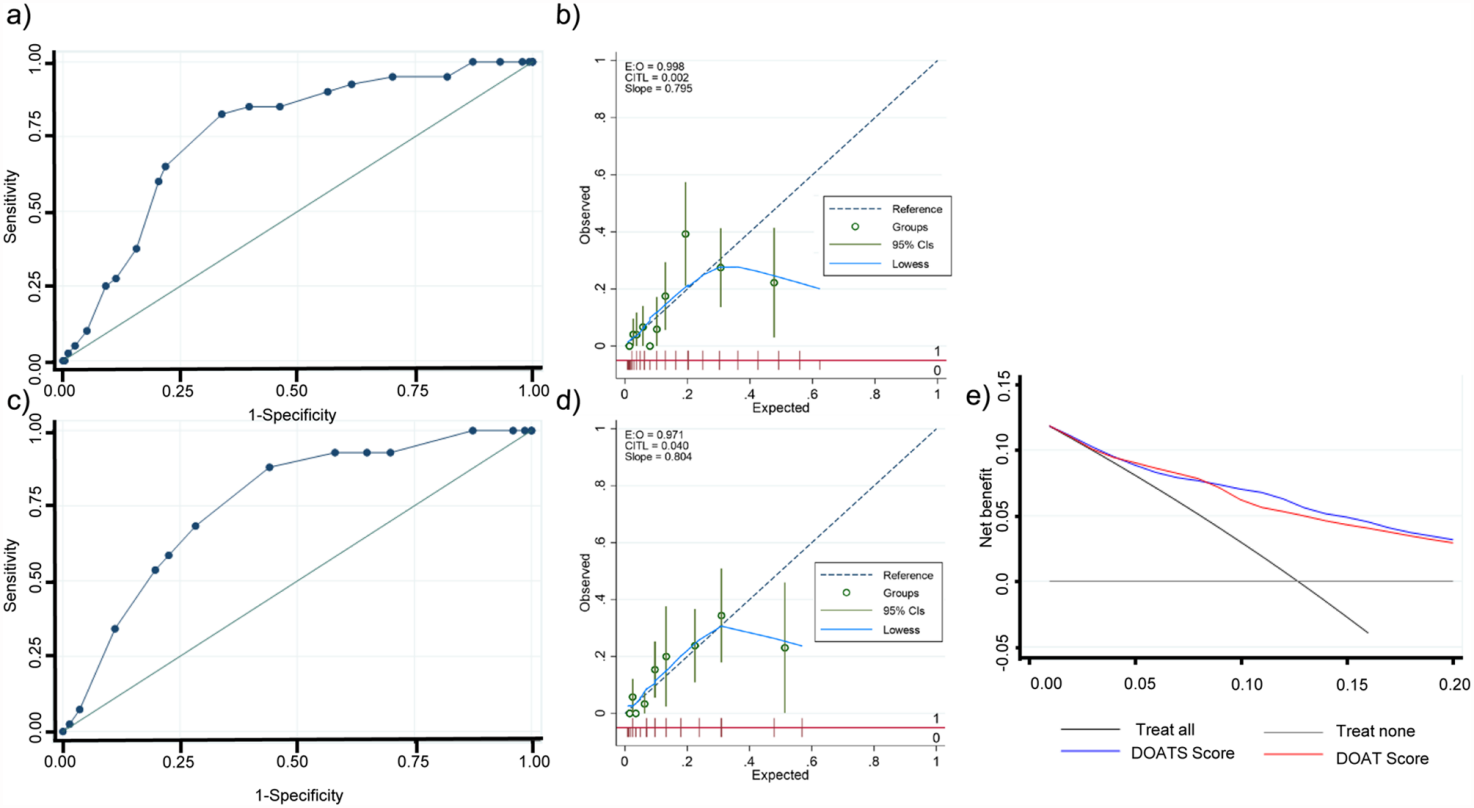
Discrimination (**a**) and calibration (**b**) of the DOATS score, discrimination (**c**) and calibration (**d**) of the DOAT score, and decision curve analyses for both scores (**e**) in the validation cohort. In the validation cohort, the area under the ROC curves (AUROCs) for (**a**) the DOATS score and (**c**) the DOAT score predicting the deterioration of COVID-19 patients during hospitalization were both 0.76 (95% CI 0.69–0.83). The calibration curve analysis revealed that the calibration slope and calibration-in-the-large were 0.80 and 0.002 for the DOATS score (**b**) and 0.80 and 0.04 for the DOAT score (**d**), respectively. (**e**) The graph illustrates the net benefit relative to no treatment in any patient (‘Treat none’) using different treatment approaches. The gray line represents the scenario where no patients are treated, resulting in a net benefit of zero (no true-positive and no false-positive classifications). The black line represents the scenario where all patients are treated. The colored lines correspond to different treatment thresholds based on the predictions of the DOATS score (blue line) and the DOAT score (red line) for the risk of deterioration. This graph demonstrates the expected net benefit when treatment decisions are made based on these different approaches. The decision curve analysis confirmed the clinical practicability of both scores in the validation cohort

The ability of these models was validated in 324 inpatients from 3 hospitals in Yamagata, Japan (Table 1). Forty-one of these patients (12.7%) experienced deterioration of their disease during hospitalization. In the validation cohort, the AUROCs of the DOATS and DOAT scores for COVID-19 deterioration were both 0.76 (95% CI 0.69–0.83) (Fig. 5a and c). Table 5 shows the percentage of patients who experienced exacerbation during hospitalization according to the scores, along with the predicted risk; although the risk was overestimated in some patients with high scores of ≥ 15 on the DOATS or ≥ 11 on the DOAT, overall, the prevalence of a worsening outcome was higher in the group with higher scores than in those with lower scores. The calibration curve analysis showed that the calibration slope and calibration-in-the-large were 0.80 and 0.002 for the DOATS score and 0.80 and 0.04 for the DOAT score, respectively (Fig. 5b and d). A DCA confirmed the clinical practicability of both scores in the validation cohort (Fig. 5e).

**Table 5:** Predicted and actual risk of COVID-19 deterioration in the validation cohort according to each (**a**) DOATS score and (**b**) DOAT score

(a)				(b)			
DOATS score	No.	Predicted (%)	Actual (%)	DOAT score	No.	Predicted (%)	Actual (%)
-2	2	0.8	0	-2	4	0.9	0
-1	4	1	0	-1	7	1.2	0
0	13	1.3	0	0	24	1.7	0
1	16	1.7	0	1	52	2.5	5.8
2	17	2.2	11.8	2	14	3.5	0
3	32	2.9	0	3	19	5	0
4	25	3.7	4	4	41	6.9	4.9
5	15	4.8	6.7	5	52	9.6	15.4
6	30	6.2	6.7	6	20	13.3	20
7	18	8	0	7	10	18	20
8	17	10.2	5.9	8	32	23.9	25
9	40	12.9	17.5	9	32	31	34.4
10	6	16.2	33.3	11	8	47.9	25
11	22	20.2	40.9	12	5	56.9	20
12	16	24.9	25				
13	7	30.2	14.3				
14	17	36.2	35.3				
15	9	42.6	22.2				
16	5	49.2	20				
17	3	55.9	33.3				
18	1	62.3	0				

Coronavirus disease 2019, COVID-19; comorbidities of diabetes/obesity, age, body temperature, and oxygen saturation, DOATS; comorbidities of diabetes/obesity, age, and body temperature, DOAT

## Discussion

We examined risk factors associated with deterioration of the COVID-19 severity in 1,675 Japanese patients < 65 years old without respiratory failure. Using the identified risk factors, we further established two predictive models; one consisting of four items (comorbidity of diabetes or obesity [DO], age [A], body temperature [T], and oxygen saturation [S], the DOATS score); and another consisting of three items (diabetes or obesity [DO], age [A], and temperature [T], the DOAT score). In cases where a doctor visited patients’ homes, information about oxygen saturation was sometimes unavailable. Therefore, we felt that prediction using three items without this variable, i.e. the DOAT score, would be easier to perform than that using the DOATS score, which consists of four items.

The predictive abilities of these scores were not significantly different in the original or validation cohorts. Because neither the DOATS nor DOAT score requires laboratory testing, physicians, nurses, and public health nurses working in hospitals, clinics, and health centers can use them both to promptly assess a patient’s deterioration risk. It should be noted that the prediction scores are intended to be calculated using data obtained only on the initial visit or first day of hospitalization; the temperature and oxygen saturation after the first encounter were not used in the calculations.

The present study included a relatively large number of subjects from Fukushima Prefecture. COVID-19 is a designated “category 2 infectious disease” in Japan, and in Fukushima Prefecture, inpatient treatment was performed for even mild cases to isolate patients from uninfected persons. Many COVID-19 studies have been conducted primarily in an inpatient setting, skewing toward severe disease. In our database, not only severely ill patients but also mild and moderate cases without respiratory failure were enrolled. As a result, over 1,500 patients who did not have respiratory failure were included in the analysis.

In the present study, clinical deterioration in each patient was identified based on data regarding starting medication against COVID-19, oxygen inhalation including nasal high flow cannula, and mechanical ventilation. This information enabled us to understand the risk factors that predict COVID-19 deterioration among patients in the early phase of the disease. Most previous studies have analyzed either risk factors that predict death or mechanical ventilation used mainly in critically ill COVID-19 patients [3–8]. However, whether or not the predictive factors identified in those studies can be used to accurately identify patients with mild cases who may experience deterioration is unclear, as the statistical analyses of those studies focused only on severe outcomes, such as death or use of mechanical ventilation. Even if disease progression does not lead to such severe outcomes, progression to respiratory failure often has a significant effect on the patient. Some patients with respiratory failure may require hospitalization for a long period of time and may require long-term oxygen therapy due to the reduction in their pulmonary function. Therefore, preventing progression to respiratory failure is considered a major medical goal. To this end, it is necessary to identify patients with mild to moderate disease who are at high risk of exacerbation and provide treatment such as neutralizing antibodies or antiviral agents to those individuals as early as possible [30, 31].

Several studies have demonstrated the association between certain clinical characteristics, such as an older age, male sex, symptoms of COVID-19 (a fever, cough, fatigue, and shortness of breath), and comorbidities (hypertension, diabetes, and CKD) and the disease severity or activity [21–23]. The addition of blood tests may also increase the accuracy of prediction of disease progression [11]. However, involving blood sampling would make it difficult for medical staff to immediately evaluate patients’ risk while in their presence. In the present study, we demonstrated two simple methods for predicting the development of illness among mild/moderate patients with COVID-19 that do not require blood sampling: DOAT and DOATS scores. Tu et al. developed a model predicting the progression of COVID-19 consisting of nine items: sex, age, and the presence of a fever, hypertension, cardio-cerebrovascular disease, dyspnea, cough, and myalgia [14]. Their model also consisted of clinical characteristics only, with no laboratory data involved. While their model had a similar predictive ability for disease progression to ours in terms of discrimination (AUROC 0.79), ours were simpler and involved fewer variables. During the pandemic, where many patients are visiting clinics, simple and quick methods of identifying patients at risk of disease deterioration are desirable.

At present, many elderly people in Japan have been vaccinated against SARS-CoV-2 [32, 33]. Delayed vaccination for middle-aged and younger people due to a vaccine shortage resulted in a shift in the epidemic statistics from elderly individuals to middle-aged/young people during the fifth pandemic wave. We suspect that this change indicates the presence of different risk factors for COVID-19 deterioration among different groups. Previously reported risk factors may therefore not be effective for the populations who are currently at highest risk because comorbidities, such as cancer and chronic respiratory or renal diseases, are relatively uncommon in young and middle-aged people. In our cohort of patients < 65 years old without respiratory failure, the reported risk factors, such as male sex, cigarette smoking, hypertension, chronic respiratory disease, malignancies, dyslipidemia, and cardiac diseases, were not included as risk factors in the final prediction models for disease deterioration. Therefore, the risk factors for deterioration of COVID-19 may need to be reconsidered and repeatedly updated in accordance with the shift of generations who are most severely suffering from COVID-19.

The strength of the current study is that the results are considered to be generalizable because this study included inpatients from a wide range of hospitals in Fukushima that handle COVID-19 inpatient treatment. In our database, three-fourths of COVID-19 patents in Fukushima were enrolled. In addition, the robustness of the predictive ability of both scores in terms of discrimination, calibration, and clinical practicability is supported by the results from the external validation cohort analyses.

However, several limitations associated with the present study also warrant mention. First, data regarding the exact proportion of vaccinated individuals were not available. COVID-19 vaccinations only began to be administered in Japan in March 2021, such that by the end of May 2021, no vaccinations had yet been administered to people < 65 years old [32, 33]. Therefore, we believe that most of the population in this study had not been vaccinated. Second, precise data regarding the day of the onset and deterioration or treatment before deterioration were not available for any patients. There may be some differences in treatment before deterioration between the Deteriorated and Stable groups. For example, treatments such as inhaled corticosteroid and favipiravir may have affected the clinical course of the patients [34–36]. In our database, information about the timing of the prescription of these medicines was not available. Third, some patients with high DOATS or DOAT scores may be overestimated with regard to their predicted risk compared to actual risk, as presented by calibration in external validation, and thus may be overtreated. However, in the face of a potentially life-threatening disease such as severe COVID-19, we believe that missed treatment opportunities due to underestimation of risk are much less desirable than overtreatment. Finally, whether or not the risk factors identified in the present study will still be applicable to risk stratification against recent COVID-19 patients is unclear, as the current situation is markedly different from that at the time of the analyses. The present study was conducted before the dissemination of the SARS-CoV-2 vaccine to nonelderly individuals and before the surge of the omicron variant. The current vaccines are reportedly less effective against omicron variants than against earlier variants [37]. Therefore, even vaccinated individuals may still become infected [38]. However, in situations where many individuals are vaccinated, it is possible that new risk factors for deterioration of COVID-19 will become apparent. Because many people in Fukushima had been vaccinated by the end of the fifth wave of the pandemic, including the younger generation, the ability of both the DOATS and DOAT scores to predict deterioration may be weakened.

In future studies, we need to validate the predictive ability of both scores using the new version of the dataset, which consists of data from many vaccinated patients infected with the delta or omicron variants of coronavirus.

## Conclusion

We found that the comorbidities of diabetes or obesity, an older age, higher body temperature, and lower oxygen saturation were risk factors that were linked to disease progression in COVID-19 patients < 65 years old who did not have respiratory failure on admission. We also established two simple prediction models that can quickly and easily evaluate the risk of the patients using the sum of the points that were given according to the presence of these risk factors. We believe that these scoring methods can be used broadly in many clinics and can effectively identify high-risk patients among COVID-19 patients < 65 years old at an early phase, resulting in a reduction of disease progression by enabling treatment to be administered as soon as possible.

### Electronic supplementary material

Below is the link to the electronic supplementary material.

**Figure S1** Restricted cubic spline curves for log odds ratios of COVID-19 deterioration versus a) age and b) oxygen saturation in the original cohort. **Table S1** Number of enrolled cases and deteriorated cases at each facility in the original and validation cohort. **Table S2** Sensitivity, specificity, and positive and negative likelihood ratios in the original cohort stratified for each threshold of a) DOATS and b) DOAT scores.

## Data Availability

The data are available from the authors upon reasonable request and with permission from the corresponding authors.

## Abbreviations

CI: Confidence interval
COVID-19: Coronavirus disease 2019
DOAT: Comorbidity of diabetes/obesity age and body temperature
DOATS: Comorbidity of diabetes/obesity age body temperature and oxygen saturation
ECMO: Extracorporeal membrane oxygenation
OR: Odds ratio
SARS-CoV-2: Severe acute respiratory syndrome coronavirus 2
