# Supplementary material for: Development and external validation of the DOAT and DOATS scores: simple decision support tools to identify disease progression among nonelderly patients with mild/moderate COVID-19
Source: BMC Pulm Med. 2023 Aug 28;23:312. doi: 10.1186/s12890-023-02604-3 (PMC10463653; doi:10.1186/s12890-023-02604-3)
Supplement: Supplementary file 1 — Figure S1 Restricted cubic spline curves for log odds ratios of COVID-19 deterioration versus a) age and b) oxygen saturation in the original cohort. Table S1 Number of enrolled cases and deteriorated cases at each facility in the original and validation cohort. Table S2 Sensitivity, specificity, and positive and negative likelihood ratios in the original cohort stratified for each threshold of a) DOATS and b) DOAT scores. [file 12890_2023_2604_MOESM1_ESM.pdf]

Supplementary Figure 1. Restricted cubic spline curves for log odds ratios of COVID-19 deterioration versus a) age and b) oxygen saturation in the original cohort.

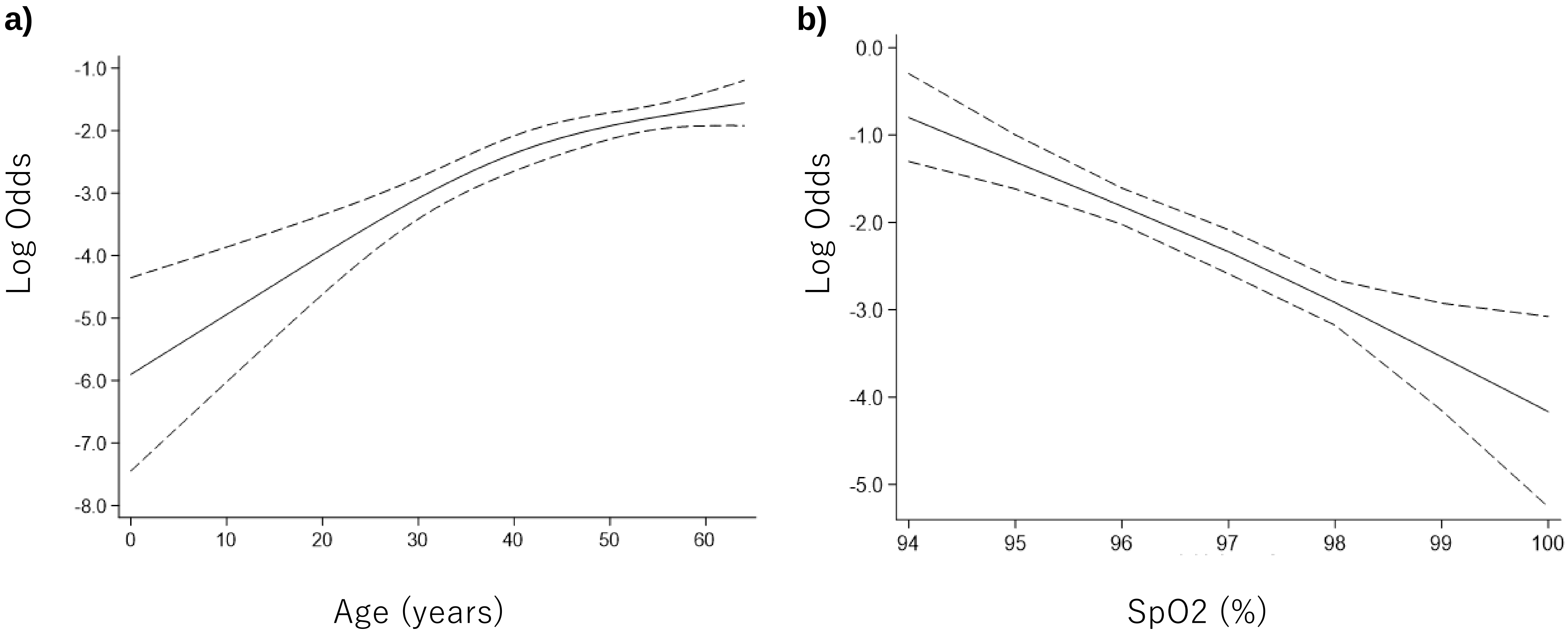

Supplementary Table 1. Number of enrolled cases and deteriorated cases at each facility in the original and validation cohort.

Original cohort		
Facility number	Deteriorated/enrolled patients	% of deteriorated patients
1	20/89	22.5
2	9/146	6.2
3	5/76	6.6
4	4/77	5.2
5	1/22	4.6
6	5/29	17.2
7	4/142	2.8
8	0/11	0
9	12/102	11.8
10	2/106	1.9
11	6/36	16.7
12	2/23	8.7
13	8/113	7.1
14	3/46	6.5
15	8/143	5.6
16	30/114	26.3
17	4/140	2.9
18	17/221	7.7
19	2/27	7.4
20	1/4	25
21	1/8	12.5
Total	144/1675	8.6

Validation cohort		
Facility number	Deteriorated/enrolled patients	% of deteriorated patients
22	17/160	10.6
23	13/89	14.7
24	11/75	14.7
Total	41/324	12.7

Supplementary Table 2. Sensitivity, specificity, and positive and negative likelihood ratios in the original cohort stratified for each threshold of a) DOATS and b) DOAT scores.
LR, likelihood ratio.

a)

Cut-off	Sensitivity	Specificity	LR (+)	LR (-)
-2	1.00	0.00	1.00	
-1	0.99	0.07	1.07	0.10
0	0.99	0.09	1.09	0.08
1	0.99	0.18	1.21	0.08
2	0.97	0.23	1.26	0.13
3	0.94	0.29	1.34	0.20
4	0.89	0.43	1.55	0.25
5	0.88	0.48	1.70	0.24
6	0.85	0.58	2.03	0.26
7	0.83	0.69	2.68	0.25
8	0.81	0.72	2.90	0.27
9	0.68	0.80	3.46	0.40
10	0.55	0.87	4.31	0.51
11	0.51	0.88	4.29	0.56
12	0.42	0.92	5.17	0.63
13	0.32	0.95	6.16	0.72
14	0.29	0.95	6.12	0.75
15	0.15	0.98	8.23	0.86
16	0.09	0.99	7.96	0.92
17	0.07	0.99	8.91	0.94
18	0.04	1.00	12.2	0.97
19	0.01	1.00	6.53	0.99

b)

Cut-off	Sensitivity	Specificity	LR (+)	LR (-)
-2	1.00	0.00	1.00	
-1	0.99	0.09	1.09	0.08
0	0.99	0.12	1.13	0.06
1	0.98	0.26	1.33	0.08
2	0.92	0.41	1.57	0.19
3	0.88	0.45	1.61	0.26
4	0.85	0.58	2.02	0.26
5	0.70	0.76	2.93	0.40
6	0.62	0.83	3.72	0.46
7	0.56	0.87	4.21	0.51
8	0.47	0.90	4.70	0.59
9	0.32	0.94	5.46	0.72
11	0.12	0.99	7.86	0.9
12	0.07	0.99	7.56	0.94
